# 

**Published:** 1869-09

**Authors:** 


VOL. XXIII. No. 9.

### THE

### Dental Register.

EDITED BY

J. TAFT & G. WATT.

SEPTEMBER, 1869.

MONTHLY:
THREE DOLLARS PER ANNUM, IN ADVANCE.

CINCINNATI:
WRIGHTSON & CO., Printers, 167 WALNUT STREET.

*J. & WM. TAFT, Dentists, 117 West Fourth St.*

